# tRNA derived fragments:A novel player in gene regulation and applications in cancer

**DOI:** 10.3389/fonc.2023.1063930

**Published:** 2023-01-20

**Authors:** Shuangshuang Zhang, Xiuchong Yu, Yaoyao Xie, Guoliang Ye, Junming Guo

**Affiliations:** ^1^ Department of Gastroenterology, The Affiliated Hospital of Medical School, Ningbo University, Ningbo, China; ^2^ Department of Biochemistry and Molecular Biology and Zhejiang Key Laboratory of Pathophysiology, School of Basic Medical Sciences, School of Medicine, Ningbo University, Ningbo, China; ^3^ Institute of Digestive Diseases, Ningbo University, Ningbo, China

**Keywords:** tRNA-derived fragments, cancer, biological role, clinical value, chemotherapy resistance

## Abstract

The heterogeneous species of tRNA-derived fragments (tRFs) with specific biological functions was recently identified. Distinct roles of tRFs in tumor development and viral infection, mediated through transcriptional and post-transcriptional regulation, has been demonstrated. In this review, we briefly summarize the current literatures on the classification of tRFs and the effects of tRNA modification on tRF biogenesis. Moreover, we highlight the tRF repertoire of biological roles such as gene silencing, and regulation of translation, cell apoptosis, and epigenetics. We also summarize the biological roles of various tRFs in cancer development and viral infection, their potential value as diagnostic and prognostic biomarkers for different types of cancers, and their potential use in cancer therapy.

## 1 Introduction

Over the past few decades, non-coding RNAs including tRNA-derived fragments (tRFs) (also named as tRNA-derived small RNAs (tsRNAs) have been verified to play crucial roles in the pathophysiological processes of cancer (1–3). Challenging the older paradigm that tRFs are merely random products of tRNAs, tRF complementarity to specific locations of pre-tRNAs or mature tRNAs demonstrates that tRFs are purposeful products of tRNAs (4). The biogenesis of tRFs is strictly controlled by a set of precise ribonucleases that produce 14–50 nucleotide-long small RNAs (5).

Evidence indicates that tRFs participate in biological processes such as gene destabilization (6, 7), mRNA processing (8), translation (9), and epigenetic regulation (10). An increasing number of studies verify that tRFs play indispensable roles in diverse diseases including cancer and viral infection (11). Here, we outline tRF biogenesis, classification, and the role of tRNA modifications on the tRF production. We also delineate the major biological functions of tRFs and the roles of tRFs in various types of cancers and viral infections. In addition, we summarize the possible usage of tRFs for cancer diagnostic and prognostic biomarkers, and as therapeutic targets. Lastly, we explore the mechanism of tRF biology that may be involved in chemoresistance.

## 2 Biogenesis and classification of tRFs

**Figure 1:**
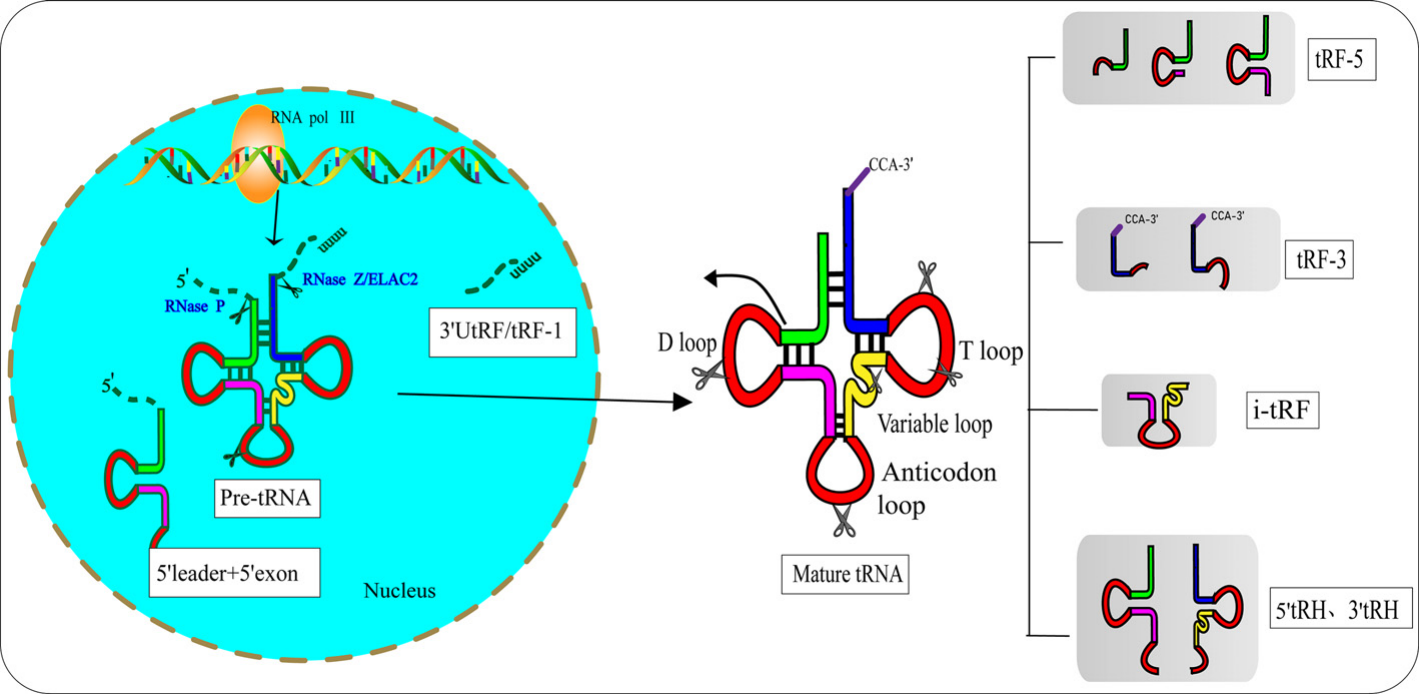
Biogenesis and classification of tRNA-derived fragments (tRFs). Pre-tRNA is transcribed by RNA Pol III in the nucleus and undergoes 5′-leader, 3′-polyU removal and 3′ CCA addition, based on disparate cleavage sites on pre-tRNA or mature tRNA. tRFs can be divided into different types including 3′U tRFs (tRF-1s), 5′-tRFs, 3′-tRFs, i′-tRFs, 5′-tRNA halves (5′-tRHs) and 3′-tRHs.

In the 1970s, researchers discovered tRFs by examining the urine of cancer patients (12). However, they did not realize that these small RNAs had biological functions. After a few decades, researchers found that tRFs are not useless debris derived from tRNAs, as they have precise sequence structures and are involved in various biological processes (13). Pre-tRNAs are produced by RNA polymerase III (RNA Pol III) located in the eukaryotic nucleus. During the maturation process of pre-tRNAs, 5′-leader and 3′-poly U nucleotides are removed by endoribonuclease P (RNase P) and ribonuclease Z (RNase Z)/cytoplasmic homolog ribonuclease Z2 (ELAC2), respectively (14, 15). The 3′-CCA tail is attached to the 3′-acceptor stem of tRNAs with the assistance of a specific tRNA nucleotide transferase (16). During the process of enzymatic splicing and chemical modifications of pre-tRNAs and mature tRNAs, tRFs are created (17). Based on the disparate cleavage sites, tRFs are classified into various distinct categories (18) (**Figure 1**): 1) 3′ U tRFs (tRF-1s), which are 16–27 nucleotides long, and are released by RNase Z or ELAC2 in the 3′-trailer sequences of pre-tRNA (19); 2) 5′-tRFs start from the 5′-termini of parental tRNAs and end at the D-loop or around the anticodon-loop; 3) tRF-3s span the 3′-termini and progress to the TΨC loop of parental tRNAs and are 18 nucleotides or 22 nucleotides in length; 4) i-tRFs (also known as tRF-2s) originate from the internal region of mature tRNAs spanning anticodons and contain D-loop and T-loop sequences, with the sequences being variable in length (13). Numerous studies have verified that the vast majority of tRFs are exclusively produced by Dicer (20, 21), while the specific ribonuclease involved in the cutting process of i-tRFs needs further elucidation; 5) tRNA halves (tiRNAs or tRHs), including 5′-tiRNAs and 3′-tiRNAs (30–40 nucleotides in length) correspond to half of a mature tRNA (22, 23). Substantial evidence has demonstrated that most tiRNAs are produced by angiogenin (ANG) under stress, such as ischemia, oxidative injury, ultraviolet exposure, arsenite exposure, or infection diseases (13, 24–27). In non-stress conditions, ANG is confined to the nucleus and exists in an inhibited state associated with the ribonuclease inhibitor RNH1. Once exposed to stress stimuli, ANG dissociates from RNH1 and translocates to the cytoplasm for tRNA processing. Sex hormone-dependent tRNA-derived RNAs are found to be abnormally expressed in non-stress conditions such as breast cancers (BCAs) with estrogen receptors (ERs) or prostate cancers (PCAs) with androgen receptors (ARs) (28). It is worth noting that ANG-dependent tiRNAs may be limited to a few tRNAs. A study identified that ANG specifically produces tiRNA^Gly^, tiRNA^Glu^, tiRNA^Lys^, tiRNA^Val^, tiRNA^His^, tiRNA^Asp^, and tiRNA^Sec^ (22). Surprisingly, the small RNA sequences from ANG-knockout cells revealed that only the abundance of tiRNA^His^ and tiRNA^Asp^ changed, suggesting that there are other ribonucleases associated with the generation of tiRNAs (29). In the ciliate *Tetrahymena* and *Saccharomyces cerevisiae*, tiRNAs are produced from the cleavage of RNase T2 family members named RNT2 and Rny1p, respectively (30, 31). All these findings have indicated the sophistication and complexity of tRFs biogenesis.

Hanada’s team discovered an atypical type of tRF that accumulate in mice with spinal motor neuron degenerative disease (32). These novel tRFs were derived from pre-tRNA^tyr^ after the aberrant removal of introns. RNA sequencing indicated that these novel tRFs encompass 5′ leader sequences starting with PPP-nucleotide and followed by 5′ exon sequences (**Figure 1**).

## 3 Effects of tRNA modification on the biogenesis of tRFs

Modified nucleotides account for 17% of all residues, and tRNAs are the most extensively modified RNAs in eukaryotes (33). Some modifications are essential for translation efficiency and the accuracy of translation through ensuring the correct wobble base pairing (34, 35), and for maintaining the stability and folding of tRNAs (36). Recently, numerous studies have shown the significance of tRNA modification in the production of tRFs (37–40).

5-methylcytosine modification is one of the important determiners in the stability of tRNAs, and can prevent tRNAs from ANG-mediated cleavage. As a result, the loss of modification leads to the degradation of tRNAs (40). Blanco et al. identified that NSun2-mediated cytosine-5 RNA methylation at the variable loop can lead to an increased affinity of tRNA and ANG, which gives rise to an accumulation of 5′-tRFs and the subsequent attenuation of protein translation rates (41). Similarly, Tuorto et al. revealed a quantitative loss of cytosine-C5 tRNA methylation in mice with DNMT2 and NSUN2 deficiencies, which led to a substantial decrease in abundance of tRNA^Asp-GTC^ and tRNA^Gly-GCC^ and reduced efficiency of overall protein synthesis (42). However, specific tRFs were not revealed by the authors. Analogously, Chen et al. developed a mouse strain with demethylase α-ketoglutarate-dependent dioxygenase alkB homolog 3 (ALKBH3) knockdown and found that ALKBH3 potently and selectively demethylated the m1A and m3C residues on tRNA. However, the expression level of the most targeted tRNAs, aside from tRNA^GlyGCC^, were not significantly changed in HeLa cells with ALKBH3 deletion (38). However, tRNA^GlyGCC^ had low levels in epididymis, testis and lung samples from mice with ALKBH3 knockdown.

Pseudouridylation also plays critical roles in regulating the abundance and species of tRFs (43). A family of pseudouridine synthases (PUS7) were reported to catalyze pseudouridylation of RNAs (44). Guzzi’s team indicated that the deficiency of PUS7 results in reduced levels of ~18 nucleotide-long 5′-tRFs but increased levels of 5′-tiRNAs, suggesting that the pseuduridylated modification on tRNAs are correlated with their differentiated endonuclease affinity and subsequent processing products (43).

Queuosine modification is uniquely detected on eukaryotic tRNA anticodon-loop containing G_34_U_35_N_36_ sequences (45). In the process of queuosine modification, guanine is substituted by queuine with the assistance of queuine tRNA-ribosyl transferase catalytic subunit 1 (QTRT1) (46). Wang et al. revealed that queuosine modification significantly protects tRNA^His^ and tRNA^Asn^ from ANG-mediated cleavage (37). These data provide new insights into how tRNA modifications affect small RNA pools. However, it is unclear how these modifications enhance tRNA stability. Thus, a detailed understanding of the biogenesis of tRFs requires more research.

## 4 Distinct biological roles of tRFs

tRFs are ubiquitous in all domains of organisms and are associated with the pathophysiological processes of various diseases (26, 47, 48). The biological functions of tRFs have been reported in recent years. Here, we summarize the main biological roles of tRFs such as in gene silencing, RNA processing, and translational, apoptotic, and epigenetic regulation in different types of diseases.

### 4.1 Gene silencing in AGO-dependent and AGO-independent mechanisms

Researchers previously treated tRFs as a distinctive type of microRNA (miRNA). It is logical to speculate that tRFs function similarly to miRNAs. For example, Green’s group detailed that tRF-3003a (derived from tRNA^CysGCA^) confers gene silencing of Janus Kinase 3 (JAK3) by ‘seed sequence’ complementarity in osteoarthritis chondrocytes (49). They further verified that tRF-3003a associated with AGO2 and GW182, and forms RNA-induced silencing complex by performing AGO2 and GW182 RNA immunoprecipitation assays. Another study found that the C-terminus of AGO2 was indispensable for functionality, but the N-terminus was not indispensable for interacting with GW182 (50). The effects of GW182 were suggested to be quite important in AGO2-mediated gene silencing. However, more research is needed to uncover the details of tRFs in AGO-dependent gene silencing.

**Figure 2:**
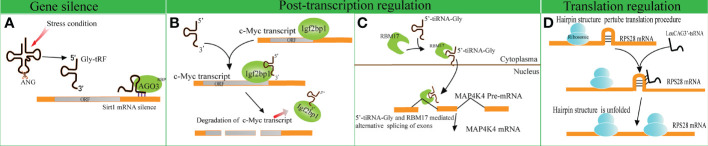
Gene expression regulation by tRNA-derived fragments (tRFs). **(A)** Gly-tRFs inhibit the expression of Sirt1 by associating with AGO3 to target Sirt1 mRNA 3′-UTR. **(B)** 5′-tsRNAs sequester RBP IGF2BP1 from the c-Myc mRNA and affects the stability of the target mRNA. **(C)** tiRNA-Gly promotes translocation of RBM17 from the cytoplasm to the nucleus, and induces RBM17-dependent splicing of MAP4K4 mRNA. **(D)** LeuCAG3’-tsRNA promotes the translation efficiency of ribosomal protein S28 (*RPS28*) mRNA by unfolding the hairpin structure of *RPS28*.

Several studies have indicated that disparate tRFs show distinct affinities with various AGO subtypes. An earlier meta-analysis revealed that several tRFs have a stronger affinity to AGO1, AGO3 and AGO4 in comparison with AGO2, indicating that tRFs possess other mechanisms of action beyond binding to AGO2, unlike miRNAs (51). A tRF named CU1276 suppresses the endogenous expression of Replication Protein1 (RPA1) by sequence complementarity, while the RNA-induced silencing complex is composed of AGO3, AGO4 and AGO1 rather than AGO2 (4, 52). Likewise, Zhong et al. identified that Gly-tRF (5′-tRF, with the length of 29–34 nucleotides) is upregulated in both ethanol-fed mice and alcoholic fatty liver disease patients. Further research has indicated that alcohol consumption can result in the activation of oxidative stress and the subsequent upregulation of Gly-tRFs (53). Gly-tRFs interact with AGO3, but not with other types of AGOs, to silence sirtuin1 (Sirt1) expression by targeting its 3′-UTR. This leads to the disruption of lipid metabolism pathways and liver injury (**Figure 2A**). However, the potential mechanism of discrepant affinity of tRFs to AGO needs further study.

### 4.2 Post transcriptional regulation with RNA binding proteins

In comparison to the studies described above, other studies support that tRFs play a role in gene silencing by interacting with RNA binding proteins (RBPs). A previous study verified that a series of i-tRFs (derived from tRNA^GluYTC^, tRNA^AspGTC^, tRNA^GlyTCC^, and tRNA^TyrGTA^) competitively bind to Y-box binding protein 1 (YBX1), known to stabilize oncogenic transcripts. The upregulation of i-tRFs sequesters YBX1 away from oncogenic mRNAs, leading to the degradation of oncogenic transcripts. Krishna et al. reported that a series of 5′-tsRNAs (including tsRNA-GlnCTG, tsRNA-GlyGCC, tsRNA-GluTTC, and tsRNA-ValCCC) modulate the states of mouse embryonic stem cells by influencing the abundance of stemness-marker c-Myc (7). Based on RNA pulldown assays and mass spectrometry, it has been verified that 5′-tsRNAs preferentially bind to RBP IGF2BP1. IGF2BP1 is known to maintain the stability of c-Myc mRNA by interacting with the coding region instability determinant. This association results in the instability of c-Myc mRNA and lowers c-Myc expression (**Figure 2B**). The study provides new insights in gene silencing at the post-transcriptional level.

A recent study identified that tiRNA-Gly promotes the proliferation and migration of papillary thyroid cancer cells by binding to RNA binding motif protein 17 (RBM17) (8), a spliceosome protein that can selectively splice mRNAs. The study revealed that the interaction of tiRNA-Gly and RBM17 suppresses ubiquitin-dependent degradation of RBM17, and facilitates the translocation of RBM17 from the cytoplasm to the nucleus. RBM17 mediates alternative exon splicing of Mitogen-Activated Protein 4 Kinase 4 (MAP4K4) pre-mRNA. Increased tiRNA-Gly induced higher levels of truncated MAP4K4 mRNA and lower levels of long variant MAP4K4 mRNA. MAP4K4 was known as a protein kinase to activate the MAPK pathway (54), though it was revealed that the two variants of MAP4K4 substantially phosphorylated the downstream proteins of the MAPK pathway, and the truncated variant showed a stronger effect than that of the long variant (**Figure 2C**). These data revealed the significance of alternative splicing in altering signal pathways mediated by tiRNA-Gly. This study provides novel insights into the role of tiRNAs in post-transcriptional gene expression regulation.

### 4.3 Regulation of translation

It has been demonstrated that the overall translation speed can be decreased by about 10% by tRFs (25). However, the underlying mechanism of the inhibitory effect of tRFs on protein translation is unclear. Ivanovet et al. reported that 5′-tiRNA^Ala^ and 5′-tiRNA^Cys^ promotes the synthesis of stress granules in a phospho-eIF2a-independent way, and disturbs the formation of the translation initiation complex by replacing the eukaryotic initiation factors from m7G-capped mRNAs (55). Further studies have clarified that the G-quadruplex-like structure (G4-motif) at the 5′-end of 5′-tiRNAs contribute to the formation of intermolecular RNA G-quadruplexes (9). These complexes can interact with the cold shock domain of translational silencer protein YBX1 (also referred as YB-1), which facilitates the assembly of stress granules and strengthens the resistance against stress (56). Mechanistic studies have indicated that RNA G-quadruplexes are necessary for replacing translational initiation factors (eIF4G/A) from mRNAs. However, YBX1 protein is not indispensable for interfering with translation-initiation complexes, but it is required in facilitating the assembly of stress granules (57). In addition, another study provided deep insights into how pseudouridylated tRFs affect translational initiation in embryogenesis and hematopoietic lineage manifestation (43). It was identified that tRF-5s are abundant in human embryonic stem cells (43). Pseudouridylation of tRF-5 at the U8 position (referred to as mTOGs) inhibits translation initiation by competitively binding to polyA binding protein-1 (PABPC1), eIF4G/A, and eIF4E.

tRFs not only disrupt translation initiation but also enhance translation efficiency. For example, the overexpression of LeuCAG3′-tsRNA significantly promotes the biogenesis of 18S rRNA (58). LeuCAG3′-tsRNA promotes the translation efficiency of *RPS28* mRNA by interacting with the coding sequence and unfolding the hairpin structure of *RPS28* mRNA. (**Figure 2D**).

Moreover, research has verified that the pre-tRNA trailer derived tRFs sequesters La/SSB in the cytoplasm and leads to the inhibition of HCV internal ribosome entry site-mediated translation (59). Certain aspects of tRF-5 play a critical role in translation silencing. For example, a conserved “GG” dinucleotide structure in tRF-5 contributes to translation inhibition regardless of the shortened length of RF-5 (60). These results pave the way for tRFs in translation regulation by interacting with RBPs or mRNAs.

### 4.4 Regulation of cellular apoptosis

Studies have disclosed the potential role of tRFs in regulating cell apoptosis. Saikia et al. verified that a series of tiRNAs perturb the formation of the apoptosome by interfering with the interaction of apoptotic protease activating factor-1 (APAF1) and cytochrome c, resulting in increased survival of mouse embryonic fibroblasts. The affinity of cytochrome c for disparate tiRNAs varies considerably, and it was shown that cytochrome c showed significantly lower affinity to tiRNA^Arg^ than to tiRNA^Ala^. However, how cytochrome c recognizes specific tRNA targets needs further research (61).

**Figure 3:**
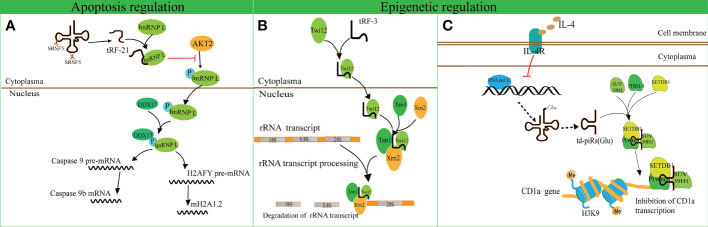
Apoptosis and epigenetic regulation by tRNA-derived fragments (tRFs). **(A)** The interaction of tRF-21-VBY9PYKHD and hnRNP L inhibits the phosphorylation of hnRNP L mediated by AKT2 and promotes the formation of the hnRNP L and DDX17 complex. This complex splices Caspase 9 and mH2A1 pre-mRNAs into Caspase 9b mRNA and mH2A1.2 mRNA. **(B)** tRF-3 translocates into the nucleus with the assistance of Twi12. The Twi12-tRF-3 complex binds to the exonuclease Xrn2 and Tan1 to form a complex, which plays important roles in rRNA processing. **(C)** IL-4 decreases the production of tRNA^Glu^ followed by the downregulation of td-piR, which assembles with PIWIL4 and recruits SETDB1, SUV39H1, and HP1β to the promoter of CD1a mRNA and facilitates methylation of H3K9 histone followed by inhibition of CD1a transcription.

A recent study revealed that tRF-21-VBY9PYKHD (i-tRF, derived from tRNA^GlyGCC^) is involved in cell apoptosis regulation (62). Inflammatory cytokine-induced tRF-21 was downregulated in pancreatic ductal adenocarcinoma (PDAC), and overexpression of tRF-21 significantly enhanced apoptosis and inhibited growth of PDAC cells. Further research revealed that tRF-21 knockdown promotes the phosphorylation of heterogeneous nuclear ribonucleoprotein L (hnRNP L) and the formation of hnRNP L and dead-box helicase 17 (DDX17) complexes. These complexes play crucial roles in splicing Caspase 9 into Caspase 9b (with anti-apoptotic specificity) and mH2A1.2 (with pro-invasive specificity), while upregulation of tRF-21 exerts the opposite effect (**Figure 3A**). These studies reveal novel apoptosis-related mechanisms for the treatment of cancers.

### 4.5 Epigenetic regulation of tRFs in a transposon-dependent manner

Transposons are genetic sequences that can translocate their sites within a genome (63). The mobilizable peculiarity of transposons is beneficial to the diversity of life and strengthens the adaptation to stress conditions (64). However, substantial evidence has shown that various cancers are significantly correlated with the transcriptional activity of transposons (65, 66). To constrain the possible harmful effects of transposons, eukaryotes have developed various mechanisms such as DNA methylation, chromatin modification, as well as RNA silencing mediated by piRNA-Piwi complexes to keep these genetic elements in a quiescent state (67). The question naturally arises of how the genome protects itself from being destructed when most of the epigenetic marks and piRNAs disappear during epigenetic reprogramming, like during embryonic development prior to implantation. It was verified that a novel class of tRF-3s (derived from mature tRNAs^LysUUU^) 18–22 nucleotides long was discovered to be enriched in SET domain bifurcated histone lysine methyltransferase 1 (SETDB1) knockout mouse embryonic stem cells (68). SETDB1 induces histone H3K9 trimethylation and plays a passive role in the transcription of long terminal repeat-retrotransposons, named endogenous retroviruses. The 18 nucleotide-long tRF-3 interferes with retroviral cDNA synthesis by displacing tRNAs from the primer binding site located in the long terminal repeat retrotransposon. The 22 nucleotide-long tRF-3 leads to the gene silencing of endogenous retrovirus mRNA through sequence complementarity to the primer binding site and results in reduced retrotransposon integration. MERVL is a retroelement that functions to drive the transcription of specific genes (69). GlyGCC 5′-tRF suppresses MERVL-mediated gene transcription by binding to heterogeneous nuclear ribonucleoproteins F and H (hnRNP F/H) to form complexes, which play a crucial role in the biogenesis of several classes of small non-coding RNAs including U7 snRNAs. The stability and utility of U7 snRNAs rely on Cajal bodies. U7 snRNAs promote the production of histone proteins by interacting with histone downstream elements. As a result, the biogenesis of histone partially halts the post-transcriptional expression of MERV-mediated genes (10).

### 4.6 Epigenetic regulation of tRFs in a Piwi-dependent manner

tRF-3 has a length of 26–31 nucleotides and has been found to interact with ribonucleoproteins AGO/Piwi and participate in epigenetic regulation (70). Couvillion et al. challenged the conventional wisdom that AGO/Piwi typically induces mRNA degradation and represses translation through RNA-induced silencing complex formation (70). They revealed that tRF-3 associates with the *Tetrahymena thermophila* AGO/Piwi protein Twi12 and promotes its nuclear translocation, while Twi12 plays essential roles in ribosomal RNA processing by assembling with Xrn2 and Tan1 proteins (**Figure 3B**). This study unveiled the roles of tRF-3s in nuclear translocation of Twi12 and possible mechanisms of epigenetic regulation. Simultaneously, it was speculated that the modified bases on tRF-3s attenuated the effects of sequence complementarity to target genes. This study may help to broaden the roles of tRF-3 and differentiate these roles from those of tRF-5.

Another study identified that tRF^Glu^ derives td-piR(Glu) with a 2′-O-methylation and 3′-terminus, and is highly enriched in monocytes in comparison to dendritic cells (71). In addition, interleukin-4 (IL-4) decreases the production of tRNA^Glu^ and its by-product td-piR(Glu) by regulating the activity of polymerase III. td-piR(Glu) functions as an IL-4-mediated signaling molecule by promoting H3K9 histone methylation, binding to PIWIL4 protein and recruiting SETDB1 and heterochromatin protein 1β (HP1β). Suppressor of variegation 3-9 homolog 1 (SUV39H1) is also recruited to the promoter of *CD1A*, which results in the suppression of *CD1A* transcriptional activity (71). These results suggest that td-piR(Glu) participates in chromatin remodeling in immune cells (**Figure 3C**).

## 5 Effect of tRFs in cancer and viral infection

Mounting evidence indicates that the dysregulation of tRFs are key players of various malignant tumors.

### 5.1 Regulation of cancer cell proliferation

Upregulated oncogenic signaling and downregulated anti-cancer signaling triggers the initiation and progression of cancers. tRF-19-3L7L73JD (i-tRF, derived from tRNA^ValAAC^) is downregulated in the plasma of pre-operative gastric cancer patients, while overexpression of tRF-19-3L7L73JD attenuates viability of gastric cancer cells by promoting cell apoptosis (72). Lee et al. demonstrated that tRF-1001 is abundantly expressed in PCA cells and the knockdown of tRF-1001 affects cell cycle distribution and reduces cell proliferation (19). In another study, 5′ tRF^HisGTG^ (derived from tRF^HisGTG^) was upregulated in colorectal cancer (CRC) tissues and positively correlated with tumor size. Moreover, the overexpression of 5′ tRF^HisGTG^ promoted cancer cell division by targeting large tumor suppressor 2 (*LATS2*), which functions in the tumor-suppressive Hippo signaling pathway (73). Analogously, tRF-Val was found to be upregulated in GC cell lines and tissues. Functionally, tRF-Val promoted proliferation of GC cells *in vivo* and *in vitro* by destabilizing the eukaryotic translation elongation gene, elongation factor 1-alpha 1 (*EEF1A1*), a regulator that mediates p53 ubiquitination by enhancing the effects of E3 ubiquitin ligase (74). This study suggests the substantial potential of tRFs in cell proliferation regulation.

### 5.2 Regulation of cancer cell migration and invasion

Migration and invasion allow cancer cells to spread to distant tissues or organs from the primary tumor site. Accumulating studies have shown that dysregulated tRFs are correlated with the invasion and metastasis of tumors. For example, Zhang et al. discovered that tRF-03357 is more abundant in ovarian cancer cells, and overexpression of tRF-03357 significantly inhibits the migration and invasion of ovarian cancer cells (75). Li et al. revealed that a cluster of 5′-tiRNAs regulate the metastatic and invasive abilities of CRC cells, and among the detected 5′- tiRNAs, 5′-tiRNA^Val^ (derived from tRNA^Val^) was verified to be positively correlated with lymph node and distant metastasis *in vivo* and *in vitro* (76). Meanwhile, another study revealed that tRF-20-MEJB5Y13 promotes the migration and invasion of CRC cells (77). Dong et al. discovered that the overexpression of tRF-24-V29K9UV3IU hinders the migratory capacity of gastric cells, and a bioinformatics analysis revealed that tRF-24-V29K9UV3IU influences signaling pathways involved in cancer metastasis (78). However, the detailed mechanism of these phenotypes requires further study.

### 5.3 Regulation of cancer cell apoptosis

Malignancy, characterized by an attenuation of cancer cell apoptosis, can also be regulated by certain tRFs. For example, overexpression of tRF-315 (derived from tRNA^lys^) inhibits the apoptosis of PCA cells by perturbing the expression of growth arrest and DNA damage 45a (*GADD45a*), which plays a vital role in sustaining *BAX* mRNA stability and facilitating the expression of the apoptotic factor *BAX* (79). These studies shed light on novel apoptosis-promoting mechanisms and could be relevant in the treatment of PCA.

### 5.4 Promotion of viral replication

A growing number of studies have identified dysregulated tRFs in cells or tissues associated with viral infection, and have revealed the function of tRFs in viral replication (11, 80–83). 5′-tRF-GlyCCC and 5′-tRF-LysCTT were discovered to be upregulated in A549 cells upon respiratory syncytial virus (RSV) infection, and overexpression of 5′-tRF-GlyCCC and 5′-tRF-LysCTT significantly promoted RSV replication (81). However, the mechanism underlying this observation was not specified in the study. Ruggero et al. demonstrated the specific function of tRF-3019 in promoting human T-celll leukemia virus type1 (HTLV-1) replication in CD4+ T cells (11). It was revealed that tRF-3019 exhibited perfect base pairing to the primer binding site of HTLV-1, and served as a primer in guiding the reverse-transcriptional activity of HTLV-1. A study by Deng et al. revealed a novel targeting mechanism of tRFs in regulating viral replication (80). For example, tRF5-GluCTC was highly abundant in airway epithelial cells upon RSV infection (80). Contrary to typical microRNAs, tRF5-GluCTC utilizes a novel gene silencing mechanism. It was reported that the interaction of the 3′-portion of and 3′-UTR of apolipoprotein E receptor 2 (*APOER2*) mRNA resulted in reduced expression of *APOER2*. APoER2 is an antiviral protein whose inhibition leads to RSV replication. Given the role of tRF5-GluCTC in promoting RSV replication (84), Choi et al. examined whether tRF5-GluCTC silences target gene expression in miRNA machinery (83). Both AGO1 and AGO4 was found to contribute to gene silencing of tRF5-GluCTC, while AGO2 and AGO3 were not involved in tRF5-GluCTC-induced gene expression activity. These observations signify the possible development of novel therapeutics against viral infection by exploiting the function of tRFs.

## 6. Clinical values of tRFs in cancers

**Figure 4:**
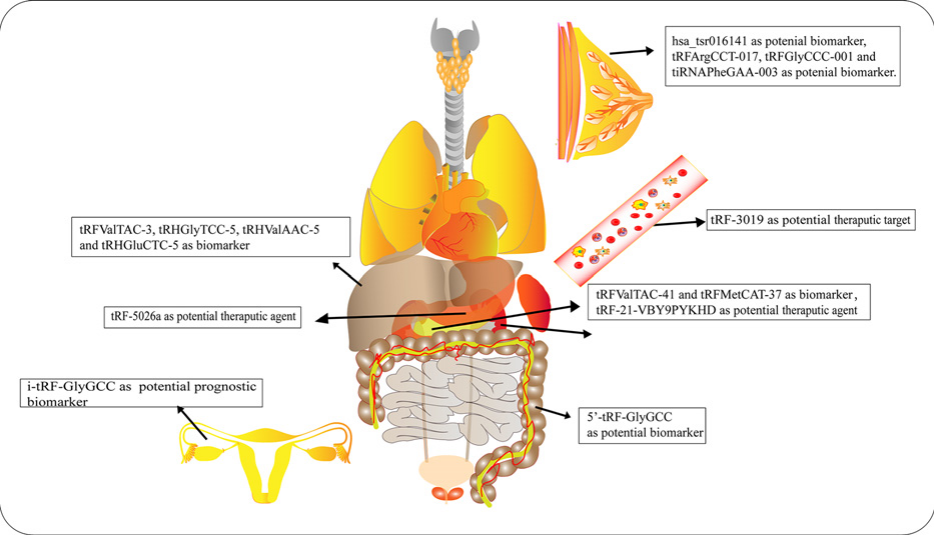
Roles of tRNA-derived fragments (tRFs) in various cancers. Different types of tRFs could serve as various biomarkers or therapeutic targets for distinct cancers or potential therapeutic agents.

Numerous studies have elucidated the abnormal expression of tRFs in various kinds of tumors and body fluids in cancer patients. There is enormous potential for tRFs to function as novel biomarkers for diagnosis, tracking the prognosis of cancer, as therapeutic targets, as well as in improving chemotherapy resistance (**Figure 4** and **Table 1**).

**Table 1:** Clinical values of tRFs in various cancers.

tRF Type	tRF name	Parental tRNA	Cancer	Expression	Source	Clinical values	Reference
tRF-5	hsa_tsr016141	tRNA-GlnTTG	BC	higher	plasma	correlated with metastasis and cancer stage and improved the diagnostic efficiency	(85)
	tRFArgCCT-017 tRFGlyCCC-001 tiRNAPheGAA-003	tRNA-ArgCCT tRNA-GlyCCC tRNA-PheGAA	BC	higher	plasma	potential diagnostic and prognostic biomarkers	(86)
	5′-tRF-GlyGCC	tRNA-GlyGCC	CRC	higher	plasma	potential diagnostic biomarker	(87)
	tRF-5026a	tRNA-ValAAC	GC	lower	tissue and plasma	potential therapeutic agent	(88)
tRF-3	tRF-3019	tRNA-ProAGG/ tRNA-ProTGG	TLL	higher	HTLV-1-infected CD4 cells	potential therapeutic target	(11)
	tRFValTAC-41/ tRFMetCAT-37	tRNA-ValTAC/ tRNA-MetCAT	PDAC	higher	serum	potential diagnostic and prognostic marker	(89)
i-tRF	i-tRF-GlyGCC	tRNA-GlyGCC	OC	higher	serum	associated with overall survival and progression free survival	(90)
	tRF-21- VBY9PYKHD	tRNA-GlyGCC	PDAC	lower	tissue	potential therapeutic agent	(62)
tRH-5	5’-tRH-GlyTCC, 5’-tRH-ValAAC, 5’- tRH-GluCTC,	tRNA- GlyTCC/ tRNA- alAAC/ tRNA- GluCTC	LC	higher	exosome from plasma	potential biomarker	(91)

### 6.1 tRFs as potential biomarkers for cancer diagnosis and prognosis

Early diagnosis and treatment are considered critical factors relating to the improved prognosis of cancer patients. Therefore, it is crucial to develop specific biomarkers to significantly improve the diagnostic efficiency for cancer patients. The fact that various types of tRFs have been detected in body fluids, such as blood, urine, saliva and sperm (92–94), has rendered tRFs promising biomarkers. Recent studies have affirmed the values of tRFs as diagnostic and prognostic markers. Panoutsopoulou et al. revealed that i-tRF-GlyGCC is abundant in ovarian cancer compared to healthy controls. A Kaplan-Meier survival analysis confirmed the diagnostic value of elevated i-tRF-GlyGCC in predicting poor overall survival (OS) and worse progression-free survival of patients (90). Another study verified that the diagnostic values of tRFArgCCT-017, tRFGlyCCC-001, and tiRNAPheGAA-003 in BCA within an area under the curve were 0.683, 0.656, and 0.666, respectively (86). Meanwhile, elevated tRFArgCCT-017 or tiRNAPhe-GAA-003 levels are correlated with worse OS and disease-free survival rates in BCA patients (86). Wu et al. revealed that the plasma levels of 5′-tRF-GlyGCC increase with the progression and metastasis of CRC (87). The combination of 5′-tRF-GlyGCC and carcinoembryonic antigen, and carbohydrate antigen 19-9 (CA19-9) improves the AUC to 0.926. Gu et al. reported that the serum level of hsa_tsr016141 is correlated with metastasis and the cancer stage of gastric cancer, and can improve the diagnostic efficiency when combined with carcinoembryonic antigen and CA19-9 (85).

Xue et al. found that the combination of tRFValTAC-41 or tRFMetCAT-37 with CA19-9 can increase the diagnostic value (AUC = 0.947 and 0.949, respectively) in PDAC compared to CA19-9 alone (AUC = 0.906), and confirmed the clinical significance of tsRNA-ValTAC-41in predicting poor OS (89). In addition, high expression of tsRNA-5001a was revealed to be associated with increased risk of postoperative recurrences and poor OS in lung adenocarcinoma (3). Moreover, several studies have revealed that tRFs can be selectively exported in extracellular vesicles (95). Zhu et al. verified that 5′-tRH-GlyTCC, 5′-tRH-ValAAC, 5′-tRH-GluCTC, and 3′-tRF-ValTAC exhibit higher levels in plasma exosomes in patients with liver cancer (91). Intriguingly, it was reported that 5′-tiRNAs can form homodimers or heterodimers to prevent endonucleolytic cleavage, thus enhancing the stability of 5′-tiRNAs in extracellular vesicles (96). The presented studies indicate the immense potential of tRFs as novel forms of bio-liquid-based markers in diagnosing and evaluating prognosis.

### 6.2 tRFs as therapeutic agents or targets for cancer therapy

In the context of tRFs with suppressive effects on tumor development, synthesized tRF mimics can be introduced into cancer cells or tissues to treat cancers. Pan et al. identified that inflammatory cytokine-induced tRF-21-VBY9PYKHD was downregulated in PDAC cells, and overexpression of tRF-21 significantly enhanced apoptosis and inhibited growth of PDAC cells (62). Mice treated with tRF-21 agomir showed a reduction in tumor volume and had longer survival times. Likewise, Zhu et al. transfected gastric cancer cells with tRF-5026a mimics and injected these cells subcutaneously into nude mice, successfully attenuating the tumor growth *in vivo* (88). Han’s group transfected CRC cells with tRF3008A (derived from tRNA^Val^) mimics and subcutaneously or intravenously injected these cells into mice to identify the effects of tRF3008A (97). tRF3008A had suppressive effects on the proliferation and migration of CRC. The results indicate the potential value of tRFs as therapeutic agents (98). In the context of oncogenic tRFs, the inhibition of certain tRFs may have therapeutic effects on cancer (99). Yang et al. demonstrated that upregulation of AS-tDR-007333 significantly promoted the growth and migration of non-small cell lung cancer cells (99). A mechanistic study revealed that AS-tDR-007333 and HSPB1 synergistically enhance transcription of mediator complex subunit 29 (*MED29*) by modifying histone modifications on *MED29* promoter regions. The therapeutic efficacy of AS-tDR-007333 was evaluated *in vivo*. Reduced levels of AS-tDR-007333 significantly inhibited growth of NSCLC tumors (99). However, it should be noted that several endogenous tRFs harbor modifications that may confer improved stability compared to synthesized tRFs.

### 6.3 Use of tRFs to improve chemotherapy resistance

**Figure 5:**
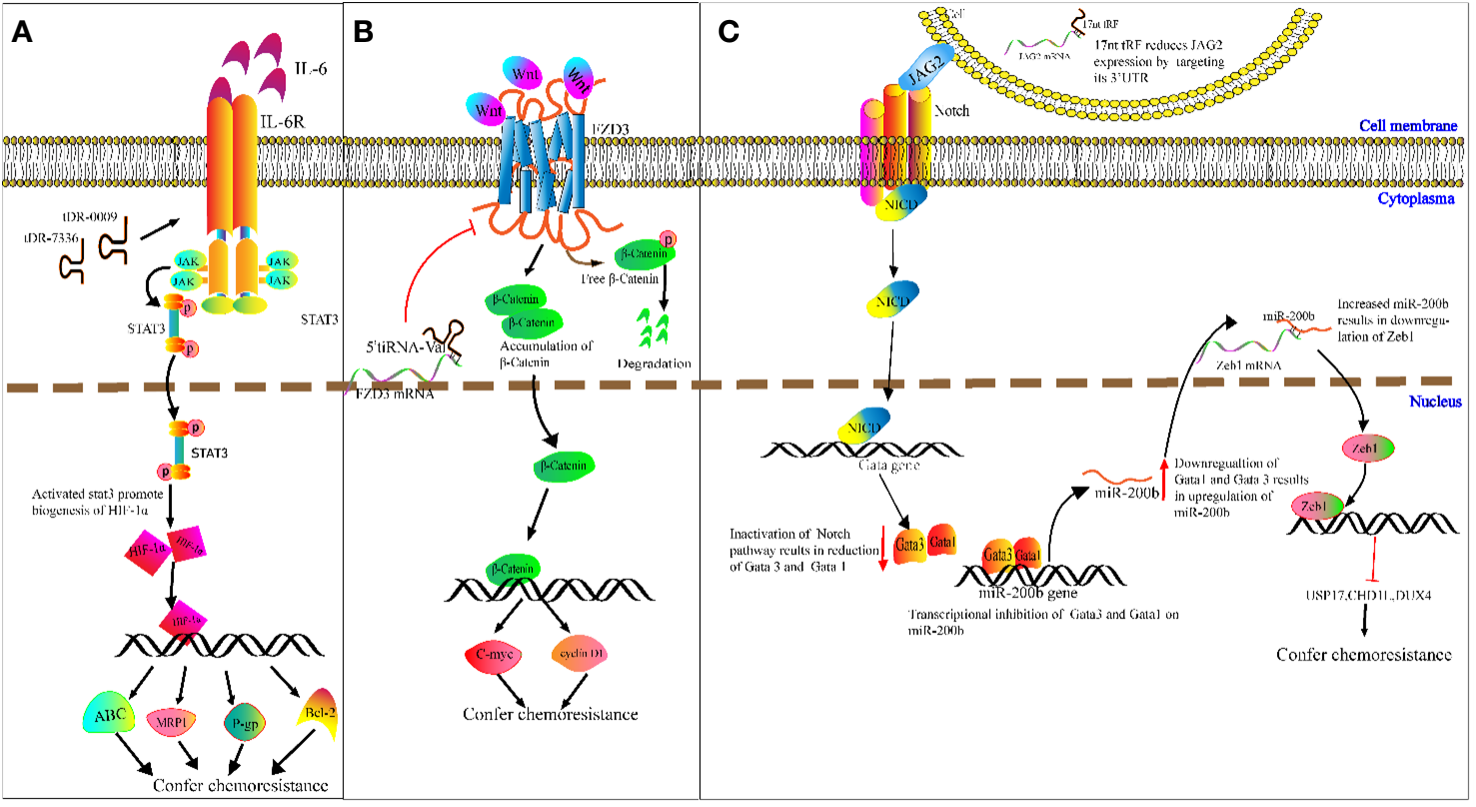
Molecular mechanism underlying tRNA-derived fragment (tRFs) regulation of carcinogenesis and chemoresistance. **(A)** DR-0009 and tDR-7336 activate the JAK/STAT3 pathway. Activated STAT3 promotes the biogenesis of HIF-1α. HIF-1α upregulates the expression of Bcl-2, ABC transporters, P-gp and MRP1. **(B)** 5′ tiRNA^Val^ suppresses FZD3/Wnt/β-Catenin signaling. Inhibition of Wnt signals results in the degradation of free β-Catenin followed by downregulation of c-Myc and cyclin D1. **(C)** The 17nt-tRF/miR-1280 suppresses Notch/JAG2 transmembrane signal transduction by downregulating JAG2. This then leads to transcriptional repression of *Gata1* and *Gata3* and upregulation of miR-200b. miR-200b downregulates ZEB1 by targeting its 3′-UTR, followed by reduced expression of USP17, CHD1L and DUX4.

Chemotherapy resistance is a challenge in cancer treatment and affects patient survival directly (100). It is therefore imperative to ascertain the potential mechanisms of cancer chemoresistance. We speculate on the potential methods that tRFs could be involved in cancer chemoresistance. For example, Cui et al. determined that tDR-0009 (derived from tRNA^GlyGCC-1-1^) and tDR-7336 (derived from tRNA^GlyGCC-1-2^) were significantly upregulated in hypoxic BCA cells, and a bioinformatics analysis revealed the two upregulated tRFs were mainly involved in cellular response to IL-6 in TNBC (101). IL-6 was reported to promote transcription of *HIF1A* by activating STAT3 signaling, and enhancing cisplatin resistance of ovarian cancer cells both *in vitro* and vivo by upregulation of *HIF1A* (102). As a transcription factor, HIF-1α induces paclitaxel and cisplatin resistance of BCA cells by increasing transcription of apoptosis-resistant Bcl-2, as well as ATP-binding cassette (ABC) transport proteins, P-glycoproteins (P-gps) and multidrug-resistant protein 1s (MRP1s) (103). On the basis of the above studies, we speculate that specific tRFs may act as new classes of regulators in cancer chemoresistance (**Figure 5A**).

Meanwhile, Mo et al. demonstrated that overexpressed 5′ tiRNA^Val^ suppresses cell proliferation and migration of BCA cells by targeting frizzled-3 (Fz-3) (104). As a vital component of the Wnt/β-catenin pathway, Fz-3 upregulation promotes expression of c-Myc, while Fz-3 inhibition results in degradation of free β-catenin (104) (105). This results in the subsequent attenuation of c-Myc expression (**Figure 5B**). c-Myc has been reported to confer antiestrogen resistance in BCA cells (106, 107).

Moreover, Huang’s team demonstrated that the 17 nucleotide-long tRF/miR-1280 suppresses proliferation and metastasis activity by destabilizing jagged canonical Notch ligand 2 (JAG2) (108). JAG2 is a membrane-bound ligand, and the binding of JAG2 to notch receptors results in proteolysis of the Notch intracellular domain, which translocates into the nucleus and binds to the promoter of *GATA1* and *GATA3* genes. GATA1 and GATA3 proteins exert transcriptional inhibition of miR-200b. As a result, the inhibition of Notch/JAG2 signaling reduces *GATA1* and *GATA3* expression, upregulates miR-200b expression, and subsequently increases expression of *ZEB1* (target gene of miR-200b) (108). While *ZEB1* was verified in CRC cells to inhibit transcription of ubiquitin-specific peptidase 17 (*USP17*), chromodomain helicase DNA-binding protein 1-like (*CHD1L*), and double homeobox 4 (*DUX4*), knockdown of *ZEB1* improved CRC cell response to cisplatin (109). For example, 17 nucleotide-long tRF/miR-1280 mediated suppression of the Notch/JAG2 pathway and results in reduced levels of ZEB1. This may improve cisplatin sensitivity in CRC (**Figure 5C**). The regulatory mechanism that tRFs may play in chemoresistance is complicated, and is unlikely to be limited to the mechanism mentioned above. However, it is likely that tRFs may participate in this elaborate network.

## 7 Conclusions and future outlook

According to existing literature, there are more than 500 tRNAs used to transport amino acids during translation (110). Therefore, it is rational to speculate that the number of existing tRF species may exceed the current estimates. The advanced technology of RNA sequencing contributes to the detection of diverse types of tRFs. tRFs have been verified to play roles in regulating the proliferation, metastasis, invasiveness and chemoresistance of cancer cells. Their specialties in cancer tissues and plasma makes it possible for scientists to develop novel screening, diagnostic and prognostic “liquid biopsy” biomarkers, as well as treatment targets for cancers. Nevertheless, as we have only scratched the surface of the biological roles of tRFs, the availability and biological significance of the various tRFs still require further investigation.

Firstly, the clinical applications of tRFs require in-depth research. Because of the extensively modified residues on tRNAs (111), tRFs inevitably contain these modifications, and may lead to inaccurate cDNA production. Our group previously combined tRF pretreatment and qRT-PCR to quantify tRFs (88), and we also applied hairpin structure primers to achieve accurate cDNA synthesis. The conventional methods used to detect tRFs mainly include high-throughput sequencing and Northern blotting. As these methods are not suitable for large-scale clinical testing, more advanced methods are needed for clinical applications.

Secondly, as tRF research in the context of chemotherapy resistance is in its infancy, it is still challenging to fully comprehend the potential mechanisms of the role of tRFs in chemoresistance. Therefore, the depth and breadth of research on the carcinogenesis of tRFs should be further expanded, especially with chemotherapeutic resistance.

In conclusion, despite the limited knowledge about tRFs, it is obvious that tRFs exist ubiquitously in all domains of organisms and play formidable roles in different pathophysiological processes. However, in-depth studies are required before clinical applications can be performed.

## Conflicts of interest

The authors declare that the research was conducted in the absence of any commercial or financial relationships that could be construed as a potential conflict of interest.

## Author contributions

JG designed the study. SZ and JG wrote the manuscript. XY and YX contributed to the literature search. All authors revised the manuscript. All authors contributed to the article and approved the submitted version.
